# Risk Assessment of COVID-19 in the Iranian Health System

**DOI:** 10.1017/dmp.2021.168

**Published:** 2021-06-07

**Authors:** Hamidreza Khankeh, Pirhossein Kolivand, Mohammad Fathi, Hamidreza Lornejad, Masoumeh Abbasabadi-Arab

**Affiliations:** 1 University of Social Welfare and Rehabilitation Science, Tehran, Iran; 2 National Medical Emergency Organization, Ministry of Health and Medical Education, Tehran, Iran

**Keywords:** Covid-19, Risk Assessment, Health System, Emergency Management Program

## Abstract

**Objective:**

The outbreak of coronavirus disease 2019 (COVID-19) has exerted unprecedented pressure on healthcare systems throughout the world. This study was designed to develop a national health emergency management program based on risk assessment for COVID-19.

**Methods:**

Mixed-methods research was used. Based on recommendations of the national epidemiology committee, 2 risk scenarios were used as basic scenarios for risk assessment. Two rounds of Focus Group Discussions (FGDs) were conducted between January and May 2020 with 30 representatives of the health system. The data were collected, analyzed, and integrated by the research team.

**Results:**

In the risk matrix, “**contamination of environment and individuals**” and “**burnout of medical staff**” occupied the red zone (intolerable risk). “**Defects in screening and admissions**,” “**process disruption in medical care and rehabilitation**,” “**increased mental disorders**,” “**social dissatisfaction**,” “**the decline in healthcare services**,” and “**loss of medical staff**” were identified as the orange zone (significant risk) of the matrix.

**Conclusions:**

The avoidance of environmental and individual contamination and healthcare worker burnout are the priorities in Iran. Attention to intersectoral cooperation, the involvement of non-governmental organizations and private center capacities, integration of information health systems, and developing evidence-based protocols are other measures that can improve the health system’s capacity in the response COVID-19.

The outbreak of the novel coronavirus disease 2019, called COVID-19, has had unprecedented impacts on healthcare systems around the world. COVID-19 has been labeled as a public health emergency by the World Health Organization (WHO). More than 219 countries have been affected by COVID-19 since the outbreak. Almost 144 million people have been infected, and more than 3,000,000 have died.^1^ Iran’s health system faces additional challenges in managing the pandemic. Since the outbreak of COVID-19 in Iran, extensive interventions have been taken to control this outbreak, but more coherent, efficient, and timely actions are required.

Risk assessment is the first step in risk management that can bring the best results and the most trustworthy ways of developing response plans. In the process, the nature and extent of hazards are determined by analyzing the risks and assessing the existing vulnerable conditions that, together, can potentially have an influence on people, properties, services, livelihoods, and the environment on which they depend. Quantitative and qualitative data can be used to estimate the risk levels and can provide the basis for developing more realistic plans. The WHO emphasizes that each country must assess its risks and take rapid actions on an appropriate scale to reduce both COVID-19 transmission and its social, public, and economic impacts.^2^ The STAG-IH group in WHO regularly reviews and updates the risk assessment of COVID-19 to make recommendations for developing the WHO health emergency programs. STAG-IH recommends that all countries rapidly and robustly increase their preparedness and response actions based on their national risk assessment and the 4 WHO transmission scenarios.^3^ The process of risk assessment allows for the identification, estimation, and ranking of risks. Risk assessment not only limited to the prevention and mitigation phase of the disaster life cycle; but also be applied in the whole process including preparedness, response, and recovery, especially in cascade events.^4^ The approaches of biological hazard risk assessment consist of strategic risk assessment (risk management planning using a focus on prevention and preparedness, surge capacity, and monitoring and assessing medium- and long-term risk), rapid risk assessment (determine the level of associated risk respond to detected events and define interventions), and postevent evaluation (recovery planning, updating, and strengthening the overall risk management system).^5^ This study was designed to rapidly assess the risk of COVID-19 to the Iranian health system and to determine priority risks and interventions.

## Methods

The mixed-method approach, using both qualitative and quantitative data, was used in this study. Risk assessment is a function of 3 components: hazard, vulnerability, and exposure; risk is the estimation of these 3 components. We used the national protocol for risk assessment, developed in 2014, which has been used several times in the health system.^6^

At first, the review of national documents and reports was done since the onset of the epidemic in the country through the Emergency Operations Centers system in the Ministry of Health, the patient registration system in the Ministry of Health (MCMC), daily reports of the epidemiology committee of the Ministry of Health and approvals of the National Corona Headquarters. The data available included the number of cases, the number of deaths, the number of recovered, the death ratio of the total number of patients, and demographic data of patients. To predict the behavior of the disease outbreak and the rate of exposure, we needed a foresight scenario. The epidemiological committee used the dynamic model, and estimated COVID-19 cumulative incidence from March 11 until June 20, 2020. In this scenario, the epidemic would experience a moderate growth rate early in April, and then the epidemic growth rate would slow down until June 20, 2020, with 3550 new cases per day.^7^

Various methods can be used to assess vulnerabilities and identify hazards.^8^ The use of focus group discussions (FGDs) is an appropriate methodology for risk identification in rapid risk assessment. Thus, we used 2 rounds of multidisciplinary FGDs between January and May 2020 to risk assessment in COVID-19. The participants included 30 key informants representing the different departments of the health system. Table 1 shows the characteristics of the participants in the FGD.

**Table 1.** Demographic characteristics of FGD

Demographic variables	Frequency	Occupational department	Frequency
Gender		Vice-chancellor of Health in MOH	5
Male	23	Vice-chancellor of Treatment in MOH	3
Female	7	Vice-chancellor of Education in MOH	1
Age (y)		Vice-chancellor of Nursing in MOH	1
25 to 35	3	Control of Diseases Center in MOH	2
36 to 45	15	National Emergency Organization in MOH	8
46 or older	12	Health reference laboratory in MOH	1
Level of education		Epidemiologist	2
Master of science	7	Mental Health Group in MOH	1
General practitioner	3	Medical Sciences University	4
Specialist physician	14	Armed Forces	1
PhD in disasters and emergencies	6	Municipality of Tehran	1

In the first round, the current situation of COVID-19 in-country and other countries was shared with the participants. The model and the process of the risk assessment were also explained to the participants. Then participants were divided into 3 groups (FGDs) of 10 multidisciplinary participants. Each group held its own focus discussions headed by the group secretary. Using the brainstorming method, the vulnerabilities of the health system and its risks was identified in each group. Moderators were asked to fill in risk assessment tables designed for collecting qualitative data and recorded them. The results of all 3 groups were collected, analyzed, and integrated with the research team, and finalized vulnerability and risks were identified (Table 2).

**Table 2.** Hazard and Vulnerability Assessment of Covid-19

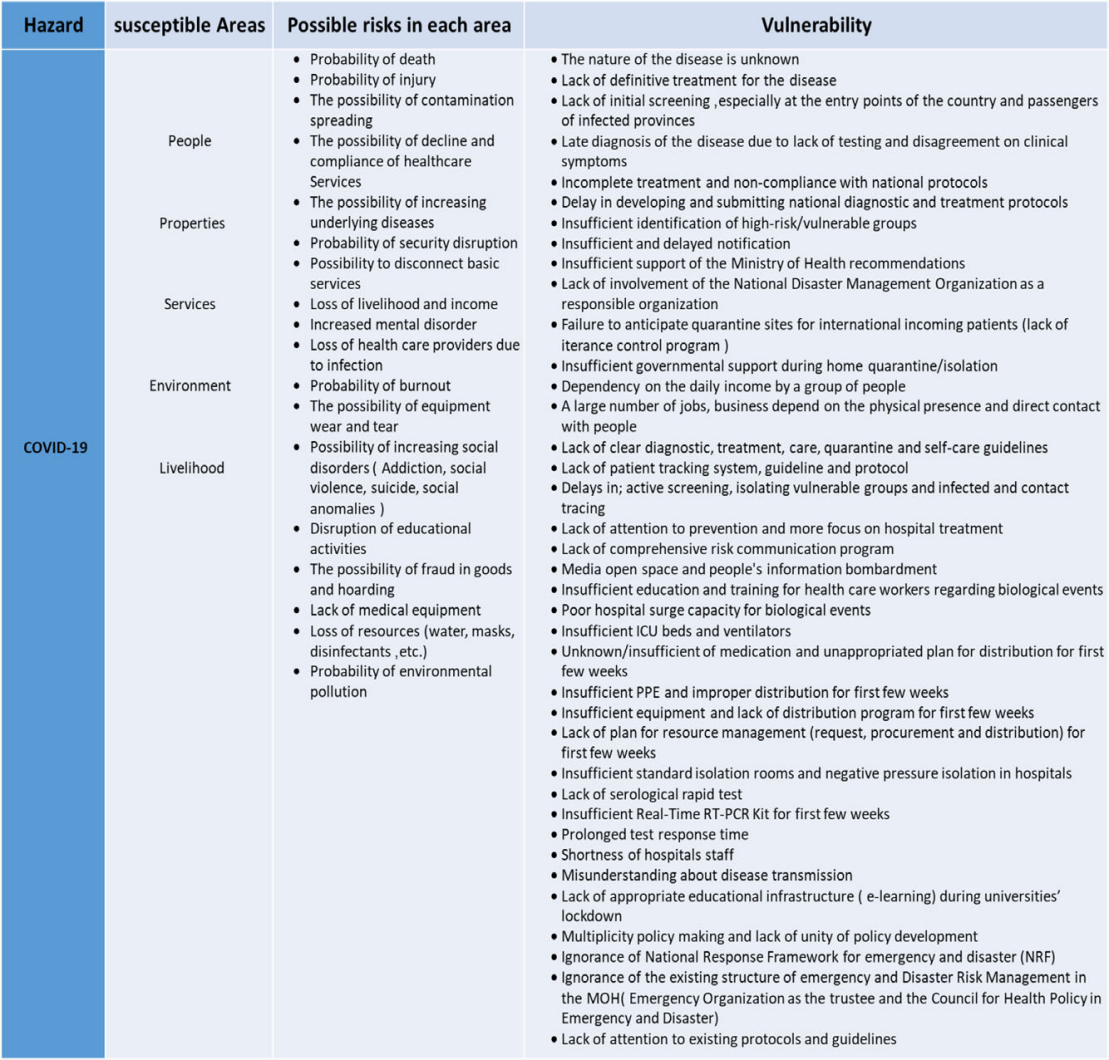

The next round of FGDs was risk scoring based on the severity and the likelihood. The participants in each group of FGDs were asked for each risk to give a score for the severity of each risk between 1 (minor) and 4 (severe) and for the likelihood of each risk between 1 (rare) and 4 (very likely). The next round of FGDs was risk scoring and was based on the severity and likelihood of occurrence. The participants in each group of FGD were asked for each risk to give a score, the severity of between 1 (minor) and 4 (severe), and for the likelihood of occurrence between 1 (rare) and 4 (very likely), and they were multiplied together to get an overall risk priority number. The risk score between 12 and 16 was considered an intolerable risk, and 8 to 9 was considered a significant risk. Intolerable and significant risks are priorities for planning. The national emergency management programs and immediate interventions to reduce the risks discuss and record in FGDs. The results of all 3 groups were collected and integrated and the final report was prepared.

## Results

In the first step of the risk assessment (determine vulnerability and exposure), participants identified the effects of COVID-19 on 5 susceptible areas (people, property, services, livelihoods, and the environment) with attention to existing vulnerabilities (Table 2).

In the second step, the risk matrix (scoring risks based on the likelihood and severity) was extracted. According to the risk matrix (Table 3), “contamination of the environment and individuals” (a score of 16) and “burnout of medical staff” (a score of 12) occupied the red zone of the matrix and identified as having the highest risk (intolerable risk). Other risks and scores were shown in Table 3.

**Table 3.** Matrix of Risk Assessment of Covid-19

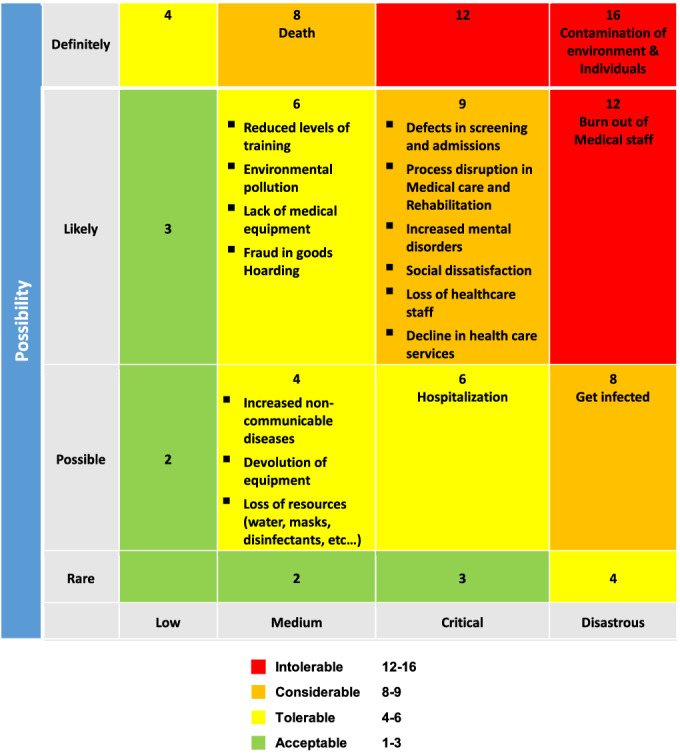

In the following step, a comprehensive national emergency management program was developed according to the capacity and vulnerabilities of the health system. These interventions were developed separately for the immediate, significant, and delayed intervention in the outbreak.

## Discussion

Pandemic or outbreak response programs in countries should be based on national risk assessments aimed at estimating the number of patients requiring hospitalization and medical equipment support. This study was designed and conducted to develop a comprehensive national emergency management program based on risk assessment for COVID-19.

According to the results of this study, the *contamination of the environment and individuals* acquired the highest score in the risk matrix and immediate intervention should be done. The health system emergency programs should be prioritized mitigating actions to reduce environmental and individual contamination. The nature of COVID-19 and the ways it was spread in the world were unknown, which led to the rapid spread of the disease in countries. The first strategy of WHO for COVID-19 is “to slow and stop transmission, to prevent outbreaks, and to delay spread.”^2^ Hence, many governments have made it their first priority to control the spread of the disease with the following measures (contact tracing; isolation or quarantine; public health measures such as hand washing, use of masks, and social distance; strengthening the prevention and infection control units in medical centers; and postponing or canceling large-scale meetings or gatherings involving a large number of people.^9^ These measures have had a major impact on disease control.^10^

*Burnout of medical staff* was on the second level in the risk matrix and immediate intervention should be planned. Healthcare providers are essential resources for the health system and their health and safety are important. Adequate, trained, experienced, responsible, and accountable staff plays an important role in achieving the goals of the health system. They need to be motivated to provide quality services to the community. Comprehensive support must be provided to maintain the health and well-being of healthcare providers. Regular and intensive training is essential for all healthcare providers improve their preparedness and effectiveness in disaster management.^11^

It is a complex process from patients *screening to treatment, and rehabilitation*. Any defect in this process will lead to an increase in disease spread and disease burden. The challenges and vulnerabilities of COVID-19 management in this respect consist of (eg, unknown/unidentified nature of the disease, its symptoms, treatment, prevention, etc., deficiencies in management and contact tracing program, deficiencies in patient flow management program, failure to manage the patient overload in hospitals and health-care centers during the first few weeks, scarcity in obtaining integrated information management (multiple databases and measurement indicators), limited access to diagnostic tests and PPE for the first few weeks, failure to identify high-risk/vulnerable groups).

*An increase in the incidence of mental disorders* during COVID-19 is an important problem in countries. Changes in lifestyle; changes in a national, organizational, or individual program; and failure to hold funerals for the comfort of the survivors of the deceased has led to increasing numbers of mental disorders. Providing stress management training services to the general and healthcare workers, paying attention to the mental health of people during quarantine, providing online and virtual psychiatric counseling services, active screening of people at risk for psychological problems and mental illness, pursuing and providing medical services to people with psychiatric disorders, providing social services and psychological support to the families of the dead people, and supporting the patient’s spouse and other relatives during the illness are effective interventions to reduce these complications.

*Social dissatisfaction* due to quarantine circumstances, loss of income and livelihood, reduced or eliminated basic services to the community, and changes of lifestyle have occurred. Identifying and providing financial support packages for vulnerable and low-income populations, economic support, and tax breaks for people are effective interventions to increase community resiliency; increasing social participation leads to improved social trust and social commitment.^12^

*Loss of medical staff* due to COVID infections and sometimes death, and or leaving the healthcare system has occurred. Developing and updating national guidelines and protocols for personnel protection, personnel time planning, volunteers and retirees management, quarantine for personnel in health centers, providing psychological first aid to employees, training and increasing the understanding of the epidemic risk of the staff, self-care, job security for health personnel, supporting medical staff, empowering and training health workers, decentralization in human resource management in times of disaster in hospitals and other health facilities and delegating authority to field managers is recommended.

**The decline in healthcare services.** People are less inclined to go to hospitals and health centers due to fear of infection/contamination, so that referrals to hospitals have been reduced, but mortality is on the rise at homes. Ignoring other health problems and other illnesses leads to an increase in silent deaths. Overconcentration of the health system on coronavirus led to the neglect of other diseases. Therefore, it is necessary to allocate some parts of the health system for these patients and any emergency interventions (eg, in the traumas) required, and all resources should not be allocated for coronavirus.

According to this study, the health system emergency programs should prioritize mitigating actions to reduce environmental and individual contamination by a virus and to manage burnout in healthcare providers. Furthermore, paying attention to inter-sectoral cooperation, involvement private hospitals and NGOs, provisioning for necessary protective equipment for patient care and treatment, paying attention to the community mental health, integrating information systems into the health system, and developing appropriate, context-bound, and evidence-based protocols are the other measures that could help improve the health system’s capacity to respond to the COVID-19 pandemic.
